# Measuring young individuals’ responses to climate change: validation of the Slovenian versions of the climate anxiety scale and the climate change worry scale

**DOI:** 10.3389/fpsyg.2023.1297782

**Published:** 2023-12-01

**Authors:** Nejc Plohl, Izidor Mlakar, Bojan Musil, Urška Smrke

**Affiliations:** ^1^Department of Psychology, Faculty of Arts, University of Maribor, Maribor, Slovenia; ^2^Faculty of Electrical Engineering and Computer Science, University of Maribor, Maribor, Slovenia

**Keywords:** climate anxiety, climate change, climate worry, psychological measurement, psychometric properties, Slovenia, validation, youth

## Abstract

**Introduction:**

While increasing awareness of climate change is needed to address this threat to the natural environment and humanity, it may simultaneously negatively impact mental health. Previous studies suggest that climate-specific mental health phenomena, such as climate anxiety and worry, tend to be especially pronounced in youth. To properly understand and address these issues, we need valid measures that can also be used in non-Anglophone samples. Therefore, in the present paper, we aimed to validate Slovenian versions of the Climate Anxiety Scale (CAS) and the Climate Change Worry Scale (CCWS) among Slovenian youth.

**Method:**

We conducted an online survey in which 442 young individuals (18–24 years) from Slovenia filled out the two central questionnaires and additional instruments capturing other relevant constructs (e.g., general anxiety, neuroticism, and behavioral engagement).

**Results:**

The confirmatory factor analyses results supported the hypothesized factorial structure of the CAS (two factors) and the CCWS (one factor). Both scales also demonstrated great internal reliability. Moreover, the analyses exploring both constructs’ nomological networks showed moderate positive associations with similar measures, such as anxiety and stress (convergent validity), and very weak associations with measures they should not be particularly related to, such as narcissism (discriminant validity). Lastly, we found that the CAS and, even more so, the CCWS have unique predictive value in explaining outcomes such as perceived threat, support for climate policies, and behavioral engagement (incremental validity).

**Discussion:**

Overall, Slovenian versions of the CAS and the CCWS seem to be valid, reliable, and appropriate for future studies tackling young individuals’ responses to climate change. Limitations of the study and areas for future research are discussed.

## 1 Introduction

Climate change is deemed to be the largest and most pervasive threat to the natural environment and humanity the world has ever experienced (United Nations, 2022), and this is something individuals worldwide are becoming increasingly aware of (Watts et al., 2019); for example, a recent survey (Poushter et al., 2022), conducted in 19 countries, showed that climate change is perceived as the number one global risk, with 75% of participants believing that it poses a major threat. While being aware of the risks imposed by climate change has a clear positive side – as it is the first step toward behaving in ways that can reduce the threat (Taylor, 2020) – it can also have negative implications for wellbeing and mental health, such as increased psychological distress, insomnia, and suicide rates (Charlson et al., 2021; Watts et al., 2021; Bingley et al., 2022; Ogunbode et al., 2023). Additionally, since climate-related stressors impact more and more people, these adverse effects on mental health are likely to become even more prominent in the future (Taylor, 2020).

Recent psychological literature has recognized the impact of environmental degradation on emotional responses, leading to the emergence of new climate-specific mental health phenomena, such as climate anxiety (also known as eco-anxiety and climate change anxiety; Albrecht, 2011; Clayton, 2020) and climate worry (also known as climate change worry; Stewart, 2021). Since these constructs have appeared in the literature only recently, reliable data regarding their prevalence is relatively lacking. However, existing data suggest that negative responses to climate change are more highly pronounced among young individuals (Clayton, 2020; Taylor, 2020). For example, a recent large-scale cross-cultural study with more than 10.000 participants aged 16–25 years reported that 84% were at least moderately worried about climate change, and more than 45% stated that their feelings about climate change negatively affected their daily functioning (Hickman et al., 2021).

Several researchers have highlighted the need for a better understanding of the predictors and consequences of such responses, as well as developing effective interventions. However, a major issue hindering these endeavors is the lack of reliable and valid instruments capable of measuring climate anxiety in different geographic and cultural contexts, particularly among high-risk groups, such as youth (Wu et al., 2020; Coffey et al., 2021). In line with this, this paper presents the Slovenian translations of the Climate Anxiety Scale (CAS; Clayton and Karazsia, 2020) and the Climate Change Worry Scale (CCWS; Stewart, 2021). Moreover, we present evidence regarding the factorial structure, reliability, and different validity aspects obtained on a sample of Slovenian youth.

## 2 Theoretical background

### 2.1 Climate anxiety and worry

While the number of studies on the psychological impact of climate change is increasing rapidly, the available literature is not entirely uniform, neither in the terms the authors employ to describe individuals’ responses to climate change nor their definitions (Coffey et al., 2021). One of the most prevalent constructs is climate anxiety, defined as the “*anxiety related to the global climate crisis and the threat of environmental disaster*” (Wu et al., 2020, p. 435) or as the “*anxiety associated with perceptions about climate change, even among people who have not personally experienced any direct impacts*” (Clayton, 2020, p. 2). As the core threat provoking this form of anxiety is realistic, it can represent an appropriate reaction and even be adaptive since it can motivate behavioral actions for addressing climate change (Clayton, 2020). In contrast, its maladaptive variation represents a psychological state that can lead to feelings of helplessness, despair, and anxious passivity (i.e., being incapable of responding to the problem in a productive capacity; Taylor, 2020), panic attacks, insomnia, obsessive thinking (Wu et al., 2020), restlessness, and sleep disturbance (Clayton and Karazsia, 2020 Crandon et al., 2022).

Another construct that has recently appeared in the literature is climate worry (Stewart, 2021). While anxiety and worry are often used interchangeably, several authors have proposed that anxiety is a more general construct that can include somatic sensations, cognitive elements, and behavioral components. In contrast, worry refers only to the cognitive content, particularly to the excessive concern about future events (Zebb and Beck, 1998). Similarly, climate worry is defined as “*primarily verbal–linguistic thoughts about the changes that may occur in the climate system and the possible effects of these changes*” (Stewart, 2021, p. 4). Such thoughts are often repetitive and challenging to control. They can be constructive, but when excessive, they may also lead to high distress and contribute to mental health problems such as anxiety (Stewart, 2021). It is worth noting that general and climate change-specific literature reports that anxiety and worry are highly correlated but may explain a unique portion of variance (Zebb and Beck, 1998; Innocenti et al., 2022).

### 2.2 Existing scales of climate anxiety and worry

Despite the growing interest regarding the psychological impacts of climate change, the number of validated scales tackling these phenomena is relatively low, with studies still often employing individual items and inventories developed for other purposes (Cruz and High, 2022). A few rare exceptions include the CAS (Clayton and Karazsia, 2020), the CCWS (Stewart, 2021), and the Hogg Eco-Anxiety Scale (Hogg et al., 2021). In the present study, we focus on the first two since CAS represents the most widely-cited (over 220 citations in SCOPUS) and translated questionnaire (at least nine language versions available), whereas CCWS was developed entirely independently and measures a related yet distinct climate change-related response (worry instead of anxiety).

The CAS was developed based on existing measures and texts describing emotional responses to climate change to create a measure that captures negative affective responses associated with awareness of climate change. The developed measure was then evaluated in two empirical studies using general population samples. The results showed that the measure has two distinct dimensions, namely cognitive and emotional impairment (rumination, difficulty sleeping or concentrating, nightmares, crying) and functional impairment due to concern about climate change (disruptions of a person’s ability to work or socialize). Moreover, the authors provided evidence regarding validity and reliability (Clayton and Karazsia, 2020). These findings were later replicated and extended by another research team (Cruz and High, 2022). So far, the measure has been translated into Filipino (Simon et al., 2022), Finnish (Niskanen, 2022), French (Mouguiama-Daouda et al., 2022), German (Wullenkord et al., 2021), Italian (Innocenti et al., 2021), Korean (Jang et al., 2023), Polish (Larionow et al., 2022), Chinese, and Japanese (Tam et al., 2023).

The CCWS was developed based on previous climate psychology and worry literature to measure the level of troubling climate change-related thoughts. The resulting measure was empirically evaluated in three studies that employed student samples. The findings showed that the CCWS is a unidimensional measure that validly and reliably captures individuals’ proximal and personal worries about climate change (Stewart, 2021). So far, the measure has been translated into Italian (Innocenti et al., 2022).

### 2.3 Theoretically expected relations and hypotheses

#### 2.3.1 Psychometric structure of the central questionnaires

The initial version of the CAS (Clayton and Karazsia, 2020) exhibited a four-factor structure, with factors representing cognitive-emotional impairment, functional impairment, behavioral engagement, and experience. For the measurement of climate anxiety, the authors proposed the first two subscales, although the assessment of a two-factor solution was not conducted in the initial study (Clayton and Karazsia, 2020; Larionow et al., 2022). There have been several adaptations of the CAS in other languages, with somewhat mixed results regarding the scale’s factorial structure (Larionow et al., 2022). In the German (Wullenkord et al., 2021) and Italian (Innocenti et al., 2021) versions, the two-factor structure did not exhibit a satisfactory fit. Instead, the authors found the one-factor structure to be superior. Other studies provided more consistent results; the French (Mouguiama-Daouda et al., 2022), Filipino (Simon et al., 2022), Chinese, Japanese, and United States versions (Tam et al., 2023) all exhibited a satisfactory fit of the two-factor solution (this solution was also superior to other tested models). One of the studies also demonstrated the configural and metric invariance of the CAS (Tam et al., 2023). Following the initial suggestion (Clayton and Karazsia, 2020) and other evidence predominantly supporting the two-factor model, we hypothesize that the Slovenian version will also consist of two factors.

*Hypothesis* 1: The CAS will demonstrate a two-factor structure, i.e., cognitive-emotional and functional impairment.

The original validation paper of the CCWS reported a one-factor solution (Stewart, 2021), which has also emerged in the Italian validation of the scale (Innocenti et al., 2022). Therefore, we hypothesize that the Slovenian version will also consist of one factor.

*Hypothesis* 2: The CCWS will demonstrate a one-factor structure.

#### 2.3.2 Convergent validity: relations among climate anxiety, worry, and related constructs

While climate anxiety and worry represent two distinct constructs, they are highly related (Stewart, 2021). Previous empirical studies suggest this is especially true for the association between cognitive-emotional impairment and climate worry, which is generally stronger than the association between functional impairment and climate worry (Innocenti et al., 2022; Tam et al., 2023).

*Hypothesis* 3: Climate anxiety and climate worry will be positively related. Additionally, we expect a higher correlation between climate worry and cognitive-emotional impairment than functional impairment.

Some definitions of climate anxiety posit it as a specific form of anxiety (Pikhala, 2020) or as an anxious response to climate change that can be clinically significant (Clayton and Karazsia, 2020). However, it also differs from other anxiety disorders as it represents an expected and adaptive response to a real threat (Hurley et al., 2022). Hence, its relation to general anxiety might not be straightforward. This is also reflected in previous studies; while some found climate anxiety to be positively related to general anxiety (Wullenkord et al., 2021), others found practically no correlation between the two (Innocenti et al., 2021; Mouguiama-Daouda et al., 2022). Similarly, mixed results were found regarding the relationship between climate worry and general anxiety. Researchers generally hypothesize that repetitive and persistent worrying can become dysfunctional to the point that it develops into a more clinically significant form or can be a symptom of already present conditions such as generalized anxiety disorder (Verplanken and Roy, 2013). This is supported by a positive correlation between the two constructs (*r* = 0.29) reported by Stewart (2021). However, a study by Innocenti et al. (2022) found no correlation between climate worry and general anxiety. Despite the previously mixed results on the association between climate anxiety, worry, and general anxiety, we follow theoretical propositions and expect a positive relationship.

*Hypothesis* 4: Climate anxiety and climate worry will be positively related to anxiety.

Previous literature implies that climate anxiety and worry may be associated with general stress. In fact, some definitions of climate anxiety understand it as climate-change-related distress (Searle and Gow, 2010), and some authors have even advocated for the use of the term “ecological stress” (Helm et al., 2018). The results of empirical studies are somewhat mixed; Stewart (2021) found a positive association between climate worry and stress, while Innocenti et al. (2022) reported a negative correlation between constructs. Nevertheless, we still expect persistent climate worrying and anxiety to be related to experiencing stress.

*Hypothesis* 5: Climate anxiety and climate worry will be positively related to stress.

Some personality traits make individuals susceptible to mental health disorders. The most prominent example is neuroticism, which is linked to the tendency to experience negative emotions and overestimate threats. It is also associated with anxiety sensitivity and could hence increase the likelihood of experiencing climate anxiety (Taylor, 2019).

*Hypothesis* 6: Climate anxiety and climate worry will be positively related to neuroticism.

Political conservatism has also been studied as a correlate of climate anxiety and worry, with the results consistently showing that it is negatively related to both constructs (Stewart, 2021; Wullenkord et al., 2021; Innocenti et al., 2022). A potential explanation could be that right-wing individuals are more prone to denying climate change, whereas climate anxiety and worry require acknowledging this phenomenon (Häkkinen and Akrami, 2014; Wullenkord et al., 2021).

*Hypothesis* 7: Climate anxiety and climate worry will be negatively related to political conservatism.

#### 2.3.3 Discriminant validity: relations to differing constructs

In exploring the discriminant validity of the CAS and the CCWS, we will first focus on narcissism. Previous studies suggest that there is either no correlation between narcissism and attitudes toward climate change (Pitiruţ et al., 2022) or that there is a negative, but very weak, association with environmental attitudes (Huang et al., 2018).

*Hypothesis* 8: Climate anxiety and climate worry will not be significantly related to narcissism.

Besides narcissism, discriminant validity will be tested by exploring the associations between climate anxiety, worry, and psychological resilience. Since this construct refers to the individual’s ability to cope with stressors (Mah et al., 2020), it is somewhat surprising that it has not yet gained much focus in climate change research. Since individuals high in psychological resilience can better navigate the stressors and adversity that climate change undoubtedly brings, there have been some discussions regarding its role in mitigating the effects of climate anxiety (Panu, 2020). Therefore, we hypothesize that individuals higher in psychological resilience will report less climate anxiety and worry but that associations will support the notion that climate anxiety and worry are different constructs than general psychological resilience.

*Hypothesis* 9: Climate anxiety and climate worry will be negatively related to psychological resilience.

#### 2.3.4 Incremental validity: relations to outcomes

Additionally, we want to assess the incremental explanatory power of climate anxiety and worry in predicting the selected outcomes, i.e., perceived threat, support for climate policies, behavioral engagement, and wellbeing.

The perceived threat imposed by climate change is closely related to climate anxiety, as climate anxiety represents a negative emotional state stemming from the perceived threat of climate change (Ogunbode et al., 2023). This is indirectly supported by previous studies that show a positive correlation between climate worry and fear of adverse weather events (Stewart, 2021) and a negative correlation between climate anxiety subdimensions and sense of safety (Larionow et al., 2022).

*Hypothesis* 10: When controlling for other relevant variables, climate anxiety and climate worry will be positively related to the perceived threat imposed by climate change.

Several previous studies (Bouman et al., 2020; Stanley et al., 2021; Wullenkord et al., 2021) found a positive association between climate anxiety and worry on the one hand and support for climate change policies on the other, potentially explained through an increased sense of responsibility when experiencing negative emotional states regarding the climate change (Bouman et al., 2020; Wullenkord et al., 2021).

*Hypothesis* 11: When controlling for other relevant variables, climate anxiety and climate worry will be positively related to the support for climate policies.

Climate anxiety is sometimes characterized as practical anxiety, as it can lead those experiencing it, similarly to climate worry, to the reassessment of their behavior and adopting a more pro-environmental stance (Hickman et al., 2021; Innocenti et al., 2021; Wullenkord et al., 2021; Innocenti et al., 2022; Larionow et al., 2022). However, not all studies found a positive relationship between climate anxiety, worry, and behavioral engagement (Clayton and Karazsia, 2020; Kapeller and Jäger, 2020). It is possible that high levels of climate anxiety and worry lead to apathy, therefore reducing the appropriate behavioral response (Kapeller and Jäger, 2020), or that by behaving pro-environmentally, individuals resolve their negative emotions regarding the perceived threat of climate change and hence report less climate anxiety and worry (Wullenkord et al., 2021). Due to the predominant support regarding a general positive relation between climate anxiety, worry, and behavioral engagement, we also expect similar results in our study.

*Hypothesis* 12: When controlling for other relevant variables, climate anxiety and climate worry will be positively related to behavioral engagement.

As the final construct in exploring the incremental validity of the two scales, we focus on psychological, social, and emotional wellbeing. Previous studies reported mixed results regarding the relationship between wellbeing, climate anxiety, and worry. Coffey et al. (2021) and Tam et al. (2023) report on the results of several studies where higher climate anxiety levels proved to be related to lower levels of psychological wellbeing (see also Wullenkord and Ojala, 2023), while a few studies, such as Reyes et al. (2021) and Ojala (2021), report no association between these constructs. Based on previous studies, we still expect climate anxiety and worry to be negatively related to psychological, social, and emotional wellbeing.

*Hypothesis* 13: When controlling for other relevant variables, climate anxiety and climate worry will be negatively related to psychological, social, and emotional wellbeing.

## 3 Methods

### 3.1 Procedure

Participants were recruited mainly through adverts posted on social media websites like Facebook and Instagram, which were targeted toward young adults living in Slovenia. The only inclusion criterion was age (i.e., individuals aged 18–24 years). After reading basic information about the study and signing the informed consent, which, among other things, described the voluntary and anonymous nature of their participation, participants completed the online assessment on a data collection website. On average, the study took about 15–20 min to complete. Participants received no compensation for filling out the questionnaire. Data were collected from November 2022 to March 2023.

### 3.2 Participants

While 737 participants started filling out the survey, some of them had to be excluded from the final sample due to prematurely dropping out (*n* = 276; 37.4%), failing to respond to more than 5.0% of items (*n* = 5; 0.7%), or not meeting our inclusion criteria regarding age (*n* = 14; 1.9%).

The final sample hence consists of 442 individuals, i.e., young adults, of whom the majority identified as female, had completed some form of secondary education prior to the study, were college students mostly studying social sciences, and described themselves as politically liberal. The detailed description of the study sample is presented in Table 1.

**Table 1:** Description of the study sample.

	*MIN* - *MAX*	*M* (*SD*)
Age	18–24	21.57 (1.67)
	*N*	%
Gender		
Female	335	75.8%
Male	98	22.2%
Other	7	1.6%
Preferred not to answer	2	0.1%
Completed education		
Primary	14	3.2%
Secondary	266	60.2%
Tertiary	162	36.7%
Status		
High school students	31	7.0%
College students	374	84.6%
Employed	26	5.9%
Other (e.g., currently unemployed)	11	2.5%
Area of study for college students		
Social sciences	174	46.5%
Natural sciences	66	17.6%
Medicine	39	10.4%
Engineering	38	10.2%
Political orientation		
Liberal		
Regarding social issues	326	73.8%
Regarding economic issues	204	46.2%
Centrist		
Regarding social issues	70	15.8%
Regarding economic issues	178	40.3%
Conservative		
Regarding social issues	46	10.4%
Regarding economic issues	60	13.6%

### 3.3 Measures

#### 3.3.1 Responses to climate change

Responses to climate change were measured with the CAS (Clayton and Karazsia, 2020) and CCWS (Stewart, 2021). CAS (Clayton and Karazsia, 2020) consists of 13 items measuring cognitive-emotional impairment (8 items, e.g., “*Thinking about climate change makes it difficult for me to sleep*”) and functional impairment (5 items, e.g., “*My concerns about climate change undermine my ability to work to my potential*”). The items are answered on a 5-point scale ranging from “*Never*” to “*Almost always*.” The subscales exhibited excellent internal consistency in the validation study (cognitive-emotional impairment: α = 0.96, functional impairment: α = 0.93). As opposed to CAS, CCWS (Stewart, 2021) consists of 10 items (e.g., “*I worry about climate change more than other people*”), answered on a 5-point scale ranging from “*Never*” to “*Always*.” The scale exhibited excellent internal consistency in the validation study (α = [0.90, 0.91]). Internal consistency coefficients for the CCWS and the CAS obtained in our study, as well as for other measures, are reported in the Results section.

#### 3.3.2 Anxiety and stress

Anxiety and stress were measured with the relevant subscales of the Depression, Anxiety, and Stress Scale (DASS-21; Lovibond and Lovibond, 1995). Each of the subscales consists of 7 items focusing on the symptoms experienced in the past week (e.g., anxiety: “*I was aware of dryness of my mouth*,” stress: “*I found it hard to wind down*”), which are answered on a 4-point scale ranging from “*Did not apply to me at all*” to “*Applied to me very much, or most of the time*.” Previous studies have shown good internal consistency of subscales (anxiety: α = [0.74, 0.83], stress: α = [0.82, 0.87]; Zanon et al., 2021).

#### 3.3.3 Neuroticism

Neuroticism was measured with the negative emotionality subscale of the Big Five Inventory-2-Short (BFI-2-S; Soto and John, 2017). The subscale consists of 6 items (e.g., “*I am someone who … worries a lot*”), which are answered on a 5-point scale ranging from “*Disagree strongly*” to “*Agree strongly*.” The subscale exhibited good internal consistency in the original validation study (α = [0.65, 0.75]).

#### 3.3.4 Political conservatism

Political conservatism was measured with two items, i.e., “*How would you describe your political outlook with regard to (1) social/(2) economic issues?*,” answered on a 7-point scale ranging from “*Very liberal*” to “*Very conservative*” (Talhelm et al., 2015). Due to the high correlation between the items, they were treated as two indicators of political conservatism (Plohl and Musil, 2022).

#### 3.3.5 Narcissism

Narcissism was measured with the Short Dark Triad questionnaire’s narcissism subscale (SD3; Jones and Paulhus, 2014). It consists of 9 items (e.g., “*People see me as a natural leader*”) answered on a 5-point scale ranging from “*Disagree strongly*” to “*Agree strongly*.” The subscale exhibited acceptable internal consistency in the validation study (α = 0.68).

#### 3.3.6 Psychological resilience

Psychological resilience was measured with the unidimensional Connor-Davidson Resilience Scale (CD-RISC; Campbell-Sills and Stein, 2007). The scale consists of 10 items (e.g., “*I am able to adapt to change*”), which are answered on a 5-point scale ranging from “*Not true at all*” to “*True nearly all the time*.” The scale exhibited great internal consistency in the validation study (α = 0.85).

#### 3.3.7 Perceived threat

The perceived threat of climate change was measured by a single-item measure of perceived threat (i.e., “*How serious of a threat is climate change to you and your family?*”; Drummond et al., 2018) that is answered on a 4-point scale ranging from “*Not at all serious*” to “*Very serious*.”

#### 3.3.8 Support for climate policies

Support for climate policies was assessed with the Resource Allocation Task (RAT), adapted from Rutjens et al. (2018), in which participants indicated their preferences regarding the distribution of Slovenia’s spending budget among 12 areas, specifically: defense, health, traffic, local and regional development, education, justice, international affairs, administration, housing assistance, general science, space and technology, social welfare, and the area of interest in this study - natural resources and the environment. Participants needed to allocate at least 1% of the budget to each area, and the distributed budget had to reach exactly 100%.

#### 3.3.9 Behavioral engagement

Behavioral engagement, i.e., engagement in pro-environmental behavior, was measured with the scale proposed by Clayton and Karazsia (2020). The scale has 6 items (e.g., “*I try to reduce my behaviors that contribute to climate change*”) answered on a 5-point scale ranging from “*Never*” to “*Almost always*.” The scale exhibited good internal consistency in its original form (α = 0.81).

#### 3.3.10 Wellbeing

Wellbeing was measured with the Mental Health Continuum Short Form (MHC-SF; Keyes et al., 2008; Lamers et al., 2011). The questionnaire consists of 14 items focusing on participants’ experiences in the past month. It measures three facets of wellbeing, i.e., emotional wellbeing (3 items, e.g., “*During the past month, how often did you feel satisfied with life?*”), social wellbeing (5 items, e.g., “*During the past month, how often did you feel that you had something important to contribute to society?*”), and psychological wellbeing (6 items, e.g., “*During the past month, how often did you feel that you had experiences that challenged you to grow and become a better person?*”). The items are answered on a 6-point scale ranging from “*Never*” to “*Everyday*.” The internal consistency of all the subscales proved to be good in previous studies (emotional wellbeing: α = 0.83, social wellbeing: α = 0.74, psychological wellbeing: α = 0.83).

The authors translated all measures that were not available in the Slovenian language, specifically the CCWS (Stewart, 2021), the CAS, and the scale measuring behavioral engagement (Clayton and Karazsia, 2020), following the back-translation procedure: original items were translated to Slovenian by one researcher, and back-translated to original language by another researcher who has not been exposed to the original items. Original and back-translated items were compared, and discrepancies were discussed until reaching an agreement, which was then reflected in the adaptations made to the Slovenian translations. Both translators had a psychology and social sciences methodology background with (close-to) native-level knowledge of both languages.

### 3.4 Statistical analyses

First, we prepared our database for analyses by excluding participants who dropped out of the study and thoroughly analyzing the share and pattern of the remaining missing values. Most of the items in the questionnaire had less than 1% of missing values, with the only exception being the RAT task (3–4% of missing values; this task was excluded from further analyses of missing values and the missing data were not imputed due to the nature of this task). In the next step, we excluded all participants with more than 5% of missing data (excluding RAT) and performed the Little’s Missing Completely at Random (MCAR) test, which showed that the remaining values can be interpreted as missing completely at random [χ^2^(3376) = 3454.17, *p* = 0.171]. These values were then imputed using the expectation–maximization algorithm based on participants’ answers to other items within the given scale.

The properties of the internal structures of the CAS and CCWS were assessed via confirmatory factor analysis (CFA) performed using R version 4.0.3 with packages *pastecs*, *Hmisc*, *MVN*, and *lavaan*. For the assessment of the model fit, the following indices were examined: Comparative Fit Index (recommended *CFI* ≥ 0.90), Tucker-Lewis Index (recommended *TLI* ≥ 0.90), Root Mean Square Error of Approximation (recommended *RMSEA* ≤ 0.08), and Standardized Root Mean Residual (recommended *SRMR* ≤ 0.08; Kline, 2005). Internal consistency of measures used was assessed via α coefficients and additionally via Guttman’s λ6 coefficients for the central questionnaires (i.e., CAS and CCWS).

The remaining analyses were performed using IBM SPSS Statistics 26. Bivariate correlations were calculated using Pearson’s correlation. Additionally, we performed 2-step hierarchical regression analyses using the “enter” method.

## 4 Results

### 4.1 Psychometric properties of the central questionnaires

#### 4.1.1 The climate anxiety scale

The Kaiser-Meyer-Olkin measure showed sampling adequacy with KMO = 0.94 and the KMO values of individual items being >0.89 (Field et al., 2012), while Bartlett’s test of sphericity χ^2^(78) = 3855.64, *p* < 0.001 indicated that item correlations were sufficiently large (Field et al., 2012). The CFA was performed to assess the 2-factor solution. Since Henze-Zirkler’s multivariate normality test indicated the absence of multivariate normality of items (*HZ* = 20.576, *p* < 0.001), we applied the unweighted least squares (ULS) estimator (Li, 2016).

The 2-factor model fit well with the observed data: *CFI* = 0.997, *TLI* = 0.996, *RMSEA* = 0.032 (90% *CI* = [0.015, 0.045]), *SRMR* = 0.057. Standardized factor loadings of the CAS items are presented in Table 2, along with their descriptive statistics. Internal consistencies of both subscales were great (cognitive-emotional impairment: α = 0.90, Guttman’s λ^6^ = 0.90, functional impairment: α = 0.86, Guttman’s λ^6^ = 0.87).

**Table 2:** Descriptive statistics of the CAS items and standardized factor loadings.

Item	*MIN*	*MAX*	*M*	*SD*	*S*	*K*	Factor loading	
							Factor 1	Factor 2
1. Thinking about climate change makes it difficult for me to concentrate.	1.00	5.00	1.98	0.95	0.69	−0.22	0.79	
2. Thinking about climate change makes it difficult for me to sleep.	1.00	5.00	1.58	0.85	1.52	2.08	0.77	
3. I have nightmares about climate change.	1.00	5.00	1.38	0.78	2.30	5.14	0.63	
4. I find myself crying because of climate change.	1.00	5.00	1.43	0.84	2.11	4.13	0.74	
5. I think, “why cannot I handle climate change better?”	1.00	5.00	2.34	1.27	0.53	−0.85	0.75	
6. I go away by myself and think about why I feel this way about climate change.	1.00	5.00	1.85	1.11	1.21	0.54	0.82	
7. I write down my thoughts about climate change and analyze them.	1.00	5.00	1.21	0.59	3.52	13.86	0.53	
8. I think, “why do I react to climate change this way?”	1.00	5.00	1.86	1.05	1.05	0.18	0.78	
9. My concerns about climate change make it hard for me to have fun with my family or friends.	1.00	5.00	1.45	0.79	1.90	3.55		0.80
10. I have problems balancing my concerns about sustainability with the needs of my family.	1.00	5.00	2.05	1.23	0.87	−0.42		0.71
11. My concerns about climate change interfere with my ability to get work or school assignments done.	1.00	5.00	1.42	0.81	2.13	4.45		0.78
12. My concerns about climate change undermine my ability to work to my potential.	1.00	5.00	1.49	0.90	1.90	2.88		0.79
13. My friends say I think about climate change too much.	1.00	5.00	1.47	0.97	2.22	4.20		0.74

S, Skewness; K, Kurtosis; Factor 1, Cognitive and emotional impairment; Factor 2, Functional impairment. All standardized factor loadings were statistically significant (*p* < 0.001).

#### 4.1.2 The climate change worry scale

The Kaiser-Meyer-Olkin measure implied sampling adequacy with KMO = 0.96 and all the KMO values of individual items being >0.94 (Field et al., 2012). Additionally, the Bartlett’s test of sphericity χ^2^(45) = 4303.42, *p* < 0.001 indicated that item correlations were sufficiently large (Field et al., 2012). The CFA was performed to assess the 1-factor solution. Since Henze-Zirkler’s multivariate normality test indicated the absence of multivariate normality of items (*HZ* = 3.776, *p* < 0.001), we used the ULS as the estimator (Li, 2016).

The 1-factor model demonstrated a good fit with the observed data: *CFI* = 0.999, *TLI* = 0.999, *RMSEA* = 0.035 (90% *CI* = [0.014, 0.053]), *SRMR* = 0.030. Standardized factor loadings and descriptive statistics are presented in Table 3. The internal consistency of the scale was excellent (α = 0.96, Guttman’s λ^6^ = 0.96).

**Table 3:** Descriptive statistics of the CCWS items and standardized factor loadings.

Item	*MIN*	*MAX*	*M*	*SD*	*S*	*K*	Factor loading
1. I worry about climate change more than other people.	1.00	5.00	2.50	1.20	0.31	−0.92	0.79
2. Thoughts about climate change cause me to have worries about what the future may hold.	1.00	5.00	3.10	1.27	−0.17	−0.97	0.89
3. I tend to seek out information about climate change in the media (e.g., TV, newspapers, internet).	1.00	5.00	2.37	1.18	0.54	−0.61	0.75
4. I tend to worry when I hear about climate change, even when the effects of climate change may be some time away.	1.00	5.00	3.02	1.39	−0.04	−1.28	0.90
5. I worry that outbreaks of severe weather may be the result of a changing climate.	1.00	5.00	3.42	1.33	−0.42	−0.99	0.85
6. I worry about climate change so much that I feel paralyzed in being able to do anything about it.	1.00	5.00	2.15	1.26	0.73	−0.71	0.78
7. I worry that I might not be able to cope with climate change.	1.00	5.00	2.56	1.31	0.31	−1.12	0.87
8. I notice that I have been worrying about climate change.	1.00	5.00	2.81	1.34	0.19	−1.11	0.91
9. Once I begin to worry about climate change, I find it difficult to stop.	1.00	5.00	1.93	1.13	1.10	0.32	0.80
10. I worry about how climate change may affect the people I care about.	1.00	5.00	2.91	1.36	0.08	−1.18	0.86

S, Skewness; K, Kurtosis. All standardized factor loadings were statistically significant (*p* < 0.001).

### 4.2 Convergent, discriminant, and incremental validity

To test convergent, discriminant, and incremental validity, we calculated bivariate correlations between relevant constructs and performed hierarchical regression analyses. Additional correlations with sociodemographic variables (age, education), which were negligible in strength, can be found in Supplementary materials.

#### 4.2.1 Convergent validity

Convergent validity was tested using Pearson’s correlation (Table 4). The results showed that the two climate anxiety dimensions were strongly positively intercorrelated (*r* = 0.85, *p* < 0.001), and both were also strongly positively associated with climate worry (cognitive-emotional impairment: *r* = 0.79, *p* < 0.001; functional impairment: *r* = 0.73, *p* < 0.001). Moreover, climate anxiety dimensions (cognitive-emotional impairment: *r* = −0.27, *p* < 0.001; functional impairment: *r* = −0.26, *p* < 0.001) and climate worry (*r* = −0.33, *p* < 0.001) exhibited weak to moderate negative correlations with political conservatism, respectively. Cognitive-emotional climate anxiety (*r* = 0.30, *p* < 0.001), functional climate anxiety (*r* = 0.30, *p* < 0.001), and climate worry (*r* = 0.32, *p* < 0.001) were moderately positively associated with anxiety. Similar associations were observed for stress as well (cognitive-emotional impairment: *r* = 0.30, *p* < 0.001; functional impairment: *r* = 0.31, *p* < 0.001; climate worry: *r* = 0.36, *p* < 0.001). Finally, associations between the central variables and neuroticism were positive and ranged from weak (cognitive-emotional impairment: *r* = 0.23, *p* < 0.001; functional impairment: *r* = 0.21, *p* < 0.001) to moderate (climate worry: *r* = 0.31, *p* < 0.001).

**Table 4:** Internal consistency coefficients and correlations between all measured constructs.

	α	*M* (*SD*)	1	2	3	4	5	6	7	8	9	10	11	12	13	14	15
1. Climate anxiety: cognitive-emotional^a^	0.90	1.70 (0.72)	–														
2. Climate anxiety: functional^a^	0.86	1.57 (0.77)	0.85^***^	–													
3. Climate worry	0.96	2.68 (1.10)	0.79^***^	0.73^***^	–												
4. Political conservatism	–	2.92 (1.26)	−0.27^***^	−0.26^***^	−0.33^***^	–											
5. Anxiety	0.88	1.94 (0.75)	0.30^***^	0.30^***^	0.32^***^	−0.13^**^	–										
6. Stress	0.89	2.25 (0.72)	0.30^***^	0.31^***^	0.36^***^	−0.16^**^	0.80^***^	–									
7. Neuroticism	0.84	3.17 (0.87)	0.23^***^	0.21^***^	0.31^***^	−0.23^***^	0.63^***^	0.76^***^	–								
8. Narcissism	0.75	2.81 (0.64)	0.01	−0.02	−0.09	0.14^**^	−0.12^**^	−0.13^**^	−0.31^***^	–							
9. Psychological resilience	0.88	3.53 (0.69)	−0.15^**^	−0.18^***^	−0.20^***^	0.17^***^	−0.39^***^	−0.47^***^	−0.61^***^	0.47^***^	–						
10. Perceived threat: climate change	–	2.64 (0.87)	0.42^***^	0.40^***^	0.57^***^	−0.22^***^	0.22^***^	0.21^***^	0.24^***^	−0.06	−0.10^*^	–					
11. Support for climate policies	–	10.34 (5.87)	0.32^***^	0.30^***^	0.34^***^	−0.20^***^	−0.07	−0.04	−0.02	−0.06	−0.05	0.17^***^	–				
12. Behavioral engagement	0.72	3.89 (0.64)	0.43^***^	0.36^***^	0.52^***^	−0.21^***^	0.20^***^	0.19^***^	0.16^***^	−0.05	0.02	0.41^***^	0.21^***^	–			
13. Emotional wellbeing	0.90	4.00 (1.04)	−0.12^**^	−0.15^**^	−0.17^***^	0.17^***^	−0.32^***^	−0.41^***^	−0.49^***^	0.35^***^	0.54^***^	−0.10^*^	−0.02	0.09	–		
14. Social wellbeing	0.80	3.11 (1.05)	−0.11^*^	−0.08	−0.23^***^	0.13^**^	−0.23^***^	−0.34^***^	−0.44^***^	0.27^***^	0.44^***^	−0.11^*^	−0.04	0.03	0.60^***^	–	
15. Psychological wellbeing	0.89	3.89 (1.14)	−0.04	−0.05	−0.10^*^	0.15^**^	−0.26^***^	−0.35^***^	−0.52^***^	0.44^***^	0.62^***^	−0.05	0.02	0.11^*^	0.72^***^	0.70^***^	–

^a^While we treat CAS as a two-dimensional scale, descriptive statistics for the total CAS score were *M* = 1.65, *SD* = 0.71. *p < 0.050. **p < 0.010. ***p < 0.001.

#### 4.2.2 Discriminant validity

Discriminant validity was also tested using Pearson’s correlation (Table 4). These analyses revealed that climate anxiety (cognitive-emotional impairment: *r* = 0.01, *p* = 0.815; functional impairment: *r* = −0.02, *p* = 0.735) and climate worry (*r* = −0.09, *p* = 0.055) were only negligibly associated with narcissism; none of the correlation coefficients reached the significance threshold. In contrast, we found significant but weak negative associations between psychological resilience and cognitive-emotional climate anxiety (*r* = −0.15, *p* = 0.002), functional climate anxiety (*r* = −0.18, *p* < 0.001), and climate worry (*r* = −0.20, *p* < 0.001).

#### 4.2.3 Incremental validity, outcomes

The relationships between climate anxiety, worry, and the selected climate change-related and general outcomes were first investigated using Pearson’s correlation (Table 4). The results showed that climate anxiety (cognitive-emotional impairment: *r* = 0.42, *p* < 0.001; functional impairment: *r* = 0.40, *p* < 0.001) and climate worry (*r* = 0.57, *p* < 0.001) are at least moderately positively associated with the perceived threat imposed by climate change. The central variables were also moderately positively associated with support for climate policies (cognitive-emotional impairment: *r* = 0.32, *p* < 0.001; functional impairment: *r* = 0.30, *p* < 0.001; climate worry: *r* = 0.34, *p* < 0.001) and behavioral engagement (cognitive-emotional impairment: *r* = 0.43, *p* < 0.001; functional impairment: *r* = 0.36, *p* < 0.001; climate worry: *r* = 0.51, *p* < 0.001). The correlations with more general outcomes were generally weaker. Specifically, we observed only negligible negative associations with psychological wellbeing (cognitive-emotional impairment: *r* = −0.04, *p* = 0.360; functional impairment: *r* = −0.05, *p* = 0.277; climate worry: *r* = −0.10, *p* = 0.037), negligible to weak associations with social wellbeing, which varied depending on the specific variable (cognitive-emotional impairment: *r* = −0.11, *p* = 0.022; functional impairment: *r* = −0.08, *p* = 0.001; climate worry: *r* = −0.23, *p* < 0.001), and weak negative associations with emotional wellbeing (cognitive-emotional impairment: *r* = −0.12, *p* = 0.009; functional impairment: *r* = −0.15, *p* = 0.001; climate worry: *r* = −0.17, *p* < 0.001).

We further performed hierarchical regression analyses regarding the incremental value of the CAS and the CCWS in predicting these climate change-related (Table 5) and general outcomes (Table 6). Variables used to investigate convergent validity (i.e., political conservatism, anxiety, stress, and neuroticism) were added in the first step, whereas the two CAS dimensions or the CCWS score were added in the second step. We also performed regression analyses, in which we controlled for age and education. Additional sociodemographic control variables did not affect our results (for more information, see Supplementary materials).

**Table 5:** Incremental validity of the CAS and CCWS in predicting climate change-related variables.

	Perceived threat: climate change		Support for climate policies		Behavioral engagement	
	Step 1	Step 2	Step 1	Step 2	Step 1	Step 2
Political conservatism	−0.17^***^	−0.08	−0.22^***^	−0.13^**^	−0.18^***^	−0.09^*^
Anxiety	0.14	0.07	−0.11	−0.17^*^	0.14	0.08
Stress	−0.04	−0.12	0.04	−0.03	0.05	−0.02
Neuroticism	0.15^*^	0.18^**^	−0.03	−0.01	−0.01	0.02
Climate anxiety: cognitive-emotional		0.27^***^		0.27^**^		0.40^***^
Climate anxiety: functional		0.12		0.10		−0.03
*R* ^2^	0.094	0.216	0.049	0.153	0.075	0.198
*F* ^a^	11.31^***^	19.98^***^	5.50^***^	12.72^***^	8.92^***^	17.86^***^
Δ*R*^2^		0.122		0.104		0.122
Δ*F*(2, 435)		33.91^***^		25.87^***^		33.12^***^
Political conservatism	−0.17^***^	−0.02	−0.22^***^	−0.11^*^	−0.18^***^	−0.05
Anxiety	0.14	0.08	−0.11	−0.15^*^	0.14	0.09
Stress	−0.04	−0.16^*^	0.04	−0.04	0.05	−0.06
Neuroticism	0.15^*^	0.14^*^	−0.03	−0.05	−0.01	−0.02
Climate worry		0.56^***^		0.38^***^		0.50^***^
*R* ^2^	0.094	0.338	0.049	0.167	0.075	0.269
*F* ^b^	11.31^***^	44.58^***^	5.50^***^	16.93^***^	8.92^***^	32.14^***^
Δ*R*^2^		0.244		0.118		0.194
Δ*F*(1, 436)		161.08^***^		59.64^***^		115.69^***^

Standardized betas are reported. ^a^Degrees of freedom (df1) and residuals (df2) were 4, 437 in Step 1 and 6, 435 in Step 2. ^b^Degrees of freedom (df1) and residuals (df2) were 4, 437 in Step 1 and 5, 436 in Step 2. **p < 0.050. **p < 0.010. ***p < 0.001.

**Table 6:** Incremental validity of the CAS and CCWS in predicting general wellbeing variables.

	Emotional wellbeing		Social wellbeing		Psychological wellbeing	
	Step 1	Step 2	Step 1	Step 2	Step 1	Step 2
Political conservatism	0.06	0.06	0.02	0.02	0.02	0.04
Anxiety	0.04	0.04	0.14^*^	0.14^*^	0.12	0.11
Stress	−0.12	−0.11	−0.11	−0.12	0.00	−0.01
Neuroticism	−0.42^***^	−0.42^***^	−0.44^***^	−0.44^***^	−0.59^***^	−0.59^***^
Climate anxiety: cognitive-emotional		0.12		−0.07		0.10
Climate anxiety: functional		−0.13		0.07		−0.03
*R* ^2^	0.249	0.254	0.202	0.203	0.279	0.284
*F* ^a^	36.22^***^	24.65^***^	27.57^***^	18.48^***^	42.36^***^	28.74^***^
Δ*R*^2^		0.005		0.001		0.005
Δ*F*(2, 435)		1.38		0.43		1.36
Political conservatism	0.06	0.06	0.02	−0.01	0.02	0.04
Anxiety	0.04	0.04	0.14^*^	0.15^*^	0.12	0.11
Stress	−0.12	−0.12	−0.11	−0.09	0.00	−0.01
Neuroticism	−0.42^***^	−0.42^***^	−0.44^***^	−0.44^***^	−0.59^***^	−0.60^***^
Climate worry		0.01		−0.12^*^		0.07
*R* ^2^	0.249	0.249	0.202	0.213	0.279	0.283
*F* ^b^	36.22^***^	28.92^***^	27.57^***^	23.49^***^	42.36^***^	34.44^***^
Δ*R*^2^		0.000		0.011		0.004
Δ*F*(1, 436)		0.05		5.93^*^		2.26

Standardized betas are reported. ^a^Degrees of freedom (df1) and residuals (df2) were 4, 437 in Step 1 and 6, 435 in Step 2. ^b^Degrees of freedom (df1) and residuals (df2) were 4, 437 in Step 1 and 5, 436 in Step 2. *p < 0.050. **p < 0.010. ***p < 0.001.

Both CAS and CCWS explained a significant share of variance in perceived threat, support for climate policies, and behavioral engagement over and above political conservatism, anxiety, stress, and neuroticism. Comparatively, the additional share of variance explained by the CCWS (regardless of the outcome) was larger compared to the CAS. A more detailed look reveals that cognitive-emotional climate anxiety was a significant positive predictor of perceived threat (*β* = 0.27, *p* < 0.001), support for climate policies (*β* = 0.27, *p* = 0.002), and behavioral engagement (*β* = 0.40, *p* < 0.001), whereas functional climate anxiety was not a significant predictor of these outcomes (perceived threat: *β* = 0.12, *p* = 0.126; support for climate policies: *β* = 0.10, *p* = 0.263; behavioral engagement: *β* = −0.03, *p* = 0.763) when controlling for other predictors. Climate change worry was a significant positive predictor of perceived threat (*β* = 0.56, *p* < 0.001), support for climate policies (*β* = 0.38, *p* < 0.001), and behavioral engagement (*β* = 0.50, *p* < 0.001) after controlling for other predictors.

The results showed that neither CAS nor CCWS were able to explain significant variance over and above political conservatism, anxiety, stress, and neuroticism in neither emotional nor psychological wellbeing. However, the results showed that CCWS, but not CAS, explained a significant share of variance in social wellbeing. In particular, after controlling for other predictors, cognitive-emotional climate anxiety (*β* = 0.12, *p* = 0.135) and functional climate anxiety (*β* = −0.13, *p* = 0.102) were not associated with emotional wellbeing. Similarly, they were not significant predictors of psychological wellbeing (cognitive-emotional impairment: *β* = 0.10, *p* = 0.219; functional impairment: *β* = −0.03, *p* = 0.704) nor social wellbeing (cognitive-emotional impairment: *β* = −0.07, *p* = 0.372; functional impairment: *β* = 0.07, *p* = 0.379). Furthermore, climate change worry was not significantly associated with emotional (*β* = 0.01, *p* = 0.830) and psychological (*β* = 0.07, *p* = 0.134) wellbeing, but was a significant negative predictor of social wellbeing (*β* = −0.12, *p* = 0.015) after controlling for other predictors.

### 4.3 Exploratory analyses: comparison of two scales

To explore the unique contribution of the CAS and CCWS, we performed additional exploratory analyses comparing the two scales. Specifically, we performed hierarchical regression analyses, in which variables used to investigate convergent validity (i.e., political conservatism, anxiety, stress, and neuroticism) were added in the first step, the two CAS dimensions in the second step, and CCWS in the third step (since analyses in section 4.2 suggested that CCWS has stronger predictive ability in our sample). The results of these analyses are presented in Table 7. Since results pertaining to Step 1 and Step 2 are already presented in Tables 5^7^ only contains results related to Step 3.

**Table 7:** Unique contribution of CCWS over CAS.

	Perceived threat: climate change	Support for climate policies	Behavioral engagement	Emotional wellbeing	Social wellbeing	Psychological wellbeing
	**Step 3**	**Step 3**	**Step 3**	**Step 3**	**Step 3**	**Step 3**
Political conservatism	−0.02	−0.11^*^	−0.05	0.06	0.00	0.04
Anxiety	0.09	−0.16^*^	0.09	0.04	0.14	0.11
Stress	−0.16^*^	−0.05	−0.05	−0.11	−0.10	−0.01
Neuroticism	0.13^*^	−0.03	−0.02	−0.43^***^	−0.41^***^	−0.59^***^
Climate anxiety: cognitive-emotional	−0.07	0.12	0.13	0.11	0.07	0.07
Climate-anxiety: functional	0.00	0.05	−0.12	−0.13	0.13	−0.04
Climate worry	0.61^***^	0.26^***^	0.48^***^	0.02	−0.26^***^	0.04
*R* ^2^	0.340	0.176	0.274	0.254	0.226	0.284
*F*(7, 434)	31.93^***^	12.83^***^	23.43^***^	21.10^***^	18.10^***^	24.64^***^
Δ*R*^2^	0.124	0.023	0.077	0.000	0.023	0.000
Δ*F*(1, 434)	81.49^***^	11.57^***^	45.80^***^	0.11	12.84^***^	0.28

Standardized betas are reported. *p < 0.050, **p < 0.010, ***p < 0.001.

The results obtained on our sample show that CCWS explained significant variance in perceived threat to climate change, support for climate policies, behavioral engagement, and social wellbeing, even after controlling for cognitive-emotional and functional climate anxiety. On the contrary, CCWS did not exhibit any significant added value to explaining emotional and psychological wellbeing.

## 5 Discussion

As climate change-related events pose a risk to young individuals’ mental health, there is a strong need for a better understanding of their responses to climate change, such as climate anxiety and worry, and the nomological network surrounding these constructs. To avoid faulty generalizations, an essential prerequisite of these endeavors are cross-cultural validations of existing scales. In the present study, we translated and validated the Slovenian versions of the CAS (Clayton and Karazsia, 2020) and the CCWS (Stewart, 2021) among Slovenian youth. We found evidence supporting the hypothesized factorial structure of the two questionnaires, internal reliability, and convergent, discriminant, and incremental validity.

Specifically, we first focused on evaluating the psychometric structure of both questionnaires. Results supported the hypothesized (H1) two-factor structure of the CAS with very satisfactory fit indices and factor loadings of all items. While previous literature is not completely consistent, with some studies reporting alternative solutions based on exploratory factor analyses (e.g., Innocenti et al., 2021; Wullenkord et al., 2021; Larionow et al., 2022), our results are in line with what was proposed in the original study and later empirically supported in the majority of adaptations (Clayton and Karazsia, 2020; Mouguiama-Daouda et al., 2022; Simon et al., 2022; Tam et al., 2023). Similarly, our results supported the proposed one-factor solution of the CCWS (H2) observed in previous studies (Stewart, 2021; Innocenti et al., 2022).

Next, we explored the nomological network of climate anxiety and worry. The results related to convergent validity were supportive of our hypotheses. Specifically, both dimensions of climate anxiety, but in particular cognitive-emotional impairment, were positively related to climate worry (H3), supporting previous studies (Innocenti et al., 2022; Tam et al., 2023). Similarly, correlations with other constructs were also supportive of our hypotheses, demonstrating positive associations of climate anxiety and worry with anxiety (H4), stress (H5), and neuroticism (H6), and negative associations with political conservatism (H7). A more detailed look at the strength of associations supports the notion that climate anxiety and worry often co-occur with more general negative emotional states, such as anxiety and stress, but also highlights the fact that these phenomena do not necessarily go hand in hand. Moreover, our results highlight that - like other variables in the context of climate change - climate anxiety is a complex, ideology-dependent construct.

To assess discriminant validity, we explored the associations between climate anxiety, worry, narcissism, and psychological resilience. Only very weak correlations were found between the focal variables and narcissism, supporting our hypothesis (H8). The relations of climate anxiety and worry with psychological resilience were negative and weak, supporting our hypothesis (H9) and the idea that psychological resilience could have a role in mitigating the effects of climate change on individuals’ responses (Mah et al., 2020; Panu, 2020) but that climate anxiety and worry differ from the general ability to cope with adversities.

Moreover, we explored the associations between climate anxiety, worry, and the selected outcomes. Correlation analyses revealed the expected positive associations between the central constructs and the perceived threat, support for climate policies, and behavioral engagement. Additional regression analyses revealed that climate anxiety and worry explained a significant share of variance in these outcomes above political conservatism, anxiety, stress, and neuroticism (H10-H12). The results remained the same when we additionally controlled for age and education. Interestingly, while climate change worry and cognitive-emotional impairment were significant predictors of these outcomes (after controlling for other predictors), functional impairment was not. A potential explanation of this result could be found in the idea that climate-change-related impaired functioning can paralyze the participants, potentially hindering their behavioral response (Kapeller and Jäger, 2020). Next, we also explored the associations between climate anxiety and worry and more general outcomes, i.e., the three dimensions of wellbeing. While the correlations were negative (as expected), the additional hierarchical regression analyses showed that neither of the core constructs explained a significant share of variance in emotional and psychological wellbeing above political conservatism, anxiety, stress, and neuroticism. The results were slightly different in the social wellbeing domain; climate worry was its negative predictor after controlling for other predictors, while the two factors of climate anxiety were not significant predictors. Although the results do not entirely defy expected relations (H13), it would be beneficial to explore these associations along with various coping strategies, which have previously been proposed as a potential moderating variable impacting the relationship between climate-change-related variables and wellbeing (Ojala, 2021; Wullenkord and Ojala, 2023).

As for the comparison between the scales, we generally found climate change worry to be a more consistent and stronger predictor of the selected outcomes in our study. In fact, our exploratory analyses revealed that CCWS may explain incremental variance in perceived threat, support for climate policies, behavioral engagement, and social wellbeing beyond CCAS. This could be ascribed to climate change worry being more pronounced than climate anxiety. Other studies have similarly reported relatively low levels of climate anxiety in non-clinical samples (Clayton and Karazsia, 2020; Wullenkord et al., 2021), while climate worry, which can precede and produce anxiety, seems to be more prevalent in such samples (Gana et al., 2001; Stewart, 2021). Our results hence suggest that in generally healthy samples, measuring climate worry may be somewhat more informative.

Lastly, while the prevalence of climate anxiety and climate worry in our sample cannot be directly compared to other studies (due to employing a rather specific sample), our results related to cognitive-emotional impairment are very similar to those reported in the original validation study (Clayton and Karazsia, 2020) as well as some later studies (e.g., Bratu et al., 2022, Study 1; Tam et al., 2023, Japan and the United States). In contrast, functional impairment seemed to be less pronounced in our study than in the original validation study (Clayton and Karazsia, 2020) and most later studies; in fact, the only studies reporting lower functional impairment were those conducted in Poland (Larionow et al., 2022), Japan, and the United States (Tam et al., 2023). Moreover, climate worry was more pronounced in our study than in the original validation study (Stewart, 2021). However, more cross-cultural research is needed to rigorously compare the rates of climate anxiety and worry observed in different countries.

### 5.1 Limitations and future research

Our study has certain limitations that may be addressed in future studies. First, as our study is based on self-report, it is possible that participants provided responses that they believe are socially desirable or had difficulty accurately recalling their feelings and behaviors. Second, the study took place online, making it impossible to control external factors that can disrupt participation. Third, the study employed a convenience sample of young individuals that is not perfectly representative of the broader population (e.g., in terms of gender), which may limit the generalizability of our findings. Moreover, as participation was voluntary, individuals who decided to participate in the study may differ from the broader population in other characteristics (e.g., interest in climate change). Lastly, although our results align with those observed in other validations of the central questionnaires, we did not explicitly test the language equivalence of the translated and original versions. Future studies may hence replicate and extend our study by employing a more diverse sample of young individuals, testing the measurement invariance of the Slovenian translations, and validating the questionnaires among other at-risk subgroups.

## 6 Conclusion

To conclude, our study shows that the Slovenian translations of the CAS (Clayton and Karazsia, 2020) and the CCWS (Stewart, 2021) are appropriate for use in future studies investigating young individuals’ responses to climate change, with CCWS generally having more predictive value in general, healthy samples. The availability of such measures in Slovene may fuel future research investigating the prevalence, temporal trends, protective and risk factors, as well as the effectiveness of interventions designed to reduce climate anxiety and worry. On the one hand, such studies may enrich the Slovenian literature on these phenomena. On the other hand, they may provide meaningful contributions to the international environmental psychology literature, which is still very much limited to studies conducted among Anglophone and WEIRD (Western, Educated, Industrial, Rich, and Democratic) populations (Tam and Milfont, 2020).

## Data availability statement

The raw data supporting the conclusions of this article will be made available by the authors, without undue reservation.

## Ethics statement

Ethical review and approval were not required for this study in accordance with the national and institutional guidelines (the study was cross-sectional and categorized as very low risk). All participants signed an informed consent. The studies were conducted in accordance with the local legislation and institutional requirements. The participants provided their written informed consent to participate in this study.

## Author contributions

NP: Conceptualization, Data curation, Formal analysis, Investigation, Methodology, Project administration, Writing – original draft, Writing – review & editing. IM: Conceptualization, Methodology, Supervision, Writing – review & editing. BM: Conceptualization, Methodology, Supervision, Writing – review & editing. US: Conceptualization, Data curation, Formal analysis, Investigation, Methodology, Project administration, Writing – original draft, Writing – review & editing.
