# Supplementary material for: Measuring young individuals’ responses to climate change: validation of the Slovenian versions of the climate anxiety scale and the climate change worry scale
Source: Front Psychol. 2023 Dec 1;14:1297782. doi: 10.3389/fpsyg.2023.1297782 (PMC10722263; doi:10.3389/fpsyg.2023.1297782)
Supplement: Supplementary file 1 [file Data_Sheet_1.pdf]

11 Supplementary materials

11.1 Correlations between CAS, CCWS, and sociodemographic variables

	M (*SD*)	1	2	3	4	5
1. Climate anxiety: cognitive-emotional	1.70 (0.72)	-				
2. Climate anxiety: functional	1.57 (0.77)	.85***	-			
3. Climate worry	2.68 (1.10)	.79***	.73***	-		
4. Age	21.57 (1.67)	-.04	-.04	.00	-	
5. Education	3.35 (0.79)	.03	-.01	.04	.50***	-

Notes. * $p < .050$, ** $p < .010$, *** $p < .001$.

11.2 Regression analyses with additional covariates

Table S1. Incremental validity of the CAS and CCWS in predicting climate change-related variables.

	**Perceived threat: climate change**			**Support for climate policies**			**Behavioral engagement**		
	**Step 1**	**Step 2**	**Step 3**	**Step 1**	**Step 2**	**Step 3**	**Step 1**	**Step 2**	**Step 3**
Age	-.08	-.07	-.05	.03	-.01	.01	-.03	-.03	.00
Education	.04	.06	.03	.00	.03	.00	.09	.11*	.07
Political conservatism		-.18***	-.08		-.22***	-.13**		-.20***	-.10*
Anxiety		.13	.07		-.10	-.17*		.16*	.10
Stress		-.04	-.12		.03	-.03		.04	-.03
Neuroticism		.15*	.19**		-.04	-.01		-.02	.01
Climate anxiety: cognitive-emotional			.27**			.27**			.39***
Climate anxiety: functional			.13			.10			-.02
R^2	.00	.10	.22	.00	.05	.15	.01	.09	.20
F^a	.97	7.88***	15.08***	.15	3.70***	9.50***	1.23	6.70***	13.76***
ΔR^2		.09	.12		.05	.10		.08	.12
ΔF^b		11.29***	33.18***		5.47***	25.59***		9.39***	32.08***
Age	-.08	-.07	-.07	.03	-.01	.00	-.03	-.03	-.02
Education	.04	.06	.01	.00	.03	-.01	.09	.11*	.07
Political conservatism		-.18***	-.02		-.22***	-.11*		-.20***	-.06
Anxiety		.13	.07		-.10	-.15*		.16*	.11
Stress		-.04	-.16*		.03	-.04		.04	-.07
Neuroticism		.15*	.14*		-.04	-.05		-.02	-.02
Climate worry			.56***			.38***			.49***
R^2	.00	.10	.34	.00	.05	.17	.01	.09	.27
F^c	.97	7.88***	32.16***	.15	3.70***	12.05***	1.23	6.70***	23.31***
ΔR^2		.09	.24		.05	.12		.08	.19
ΔF^d		11.29***	160.50***		5.47***	59.09***		9.39***	112.63***

Notes. Standardized betas are reported. [a] Degrees of freedom were 2, 439 in Step 1, 6, 435 in Step 2, and 8, 433 in Step 3. [b] Degrees of freedom were 4, 435 in Step 2 and 2, 433 in Step 3. [c] Degrees of freedom were 2, 439 in Step 1, 6, 435 in Step 2, and 7, 434 in Step 3. [d] Degrees of freedom were 4, 435 in Step 2 and 1, 434 in Step 3. * $p < .050$, ** $p < .010$, *** $p < .001$.

Table S2. Incremental validity of the CAS and CCWS in predicting general wellbeing variables.

	**Emotional wellbeing**			**Social wellbeing**			**Psychological wellbeing**		
	**Step 1**	**Step 2**	**Step 3**	**Step 1**	**Step 2**	**Step 3**	**Step 1**	**Step 2**	**Step 3**
Age	-.08	$-.10^{*}$	$-.10^{*}$	-.09	$-.10^{*}$	$-.11^{*}$	-.03	-.04	-.04
Education	.06	.08	.08	.02	.04	.05	.05	.08	.07
Political conservatism		.05	.05		.01	.01		.01	.03
Anxiety		.03	.04		.13	.13		.13	.12
Stress		-.12	-.11		-.11	-.11		.00	-.01
Neuroticism		$-.42^{***}$	$-.42^{***}$		$-.44^{***}$	$-.44^{***}$		$-.60^{***}$	$-.59^{***}$
Climate anxiety: cognitive-emotional			.11			-.08			.08
Climate anxiety: functional			-.12			.08			-.02
R^2	.01	.26	.26	.01	.21	.21	.00	.28	.29
F^a	1.10	25.13^{***}	19.16^{***}	1.57	19.23^{***}	14.52^{***}	.43	28.76^{***}	21.87^{***}
ΔR^2		.25	.00		.20	.00		.28	.00
ΔF^b		36.96^{***}	1.21		27.86^{***}	.52		42.85^{***}	1.13
Age	-.08	$-.10^{*}$	$-.10^{*}$	-.09	$-.10^{*}$	$-.11^{*}$	-.03	-.04	-.04
Education	.06	.08	.08	.02	.04	.05	.05	.08	.07
Political conservatism		.05	.05		.01	-.02		.01	.03
Anxiety		.03	.03		.13	.14		.13	.12
Stress		-.12	-.12		-.11	-.08		.00	-.02
Neuroticism		$-.42^{***}$	$-.42^{***}$		$-.44^{***}$	$-.44^{***}$		$-.60^{***}$	$-.60^{***}$
Climate worry			.01			$-.12^{*}$			.06
R^2	.01	.26	.26	.01	.21	.22	.00	.28	.29
F^c	1.10	25.13^{***}	21.49^{***}	1.57	19.23^{***}	17.54^{***}	.43	28.76^{***}	24.98^{***}
ΔR^2		.25	.00		.20	.01		.28	.00
ΔF^d		36.96^{***}	.02		27.86^{***}	6.09^{*}		42.85^{***}	1.92

Notes. Standardized betas are reported. [a] Degrees of freedom were 2, 439 in Step 1, 6, 435 in Step 2, and 8, 433 in Step 3. [b] Degrees of freedom were 4, 435 in Step 2 and 2, 433 in Step 3. [c] Degrees of freedom were 2, 439 in Step 1, 6, 435 in Step 2, and 7, 434 in Step 3. [d] Degrees of freedom were 4, 435 in Step 2 and 1, 434 in Step 3. $^{*}p < .050$, $^{**}p < .010$, $^{***}p < .001$.
